# Technical Brief: Subretinal injection and electroporation into adult mouse eyes

**Published:** 2008-12-05

**Authors:** Christiana J. Johnson, Lennart Berglin, Micah A. Chrenek, T.M. Redmond, Jeffrey H. Boatright, John M. Nickerson

**Affiliations:** 1 Department of Ophthalmology, Emory University, Atlanta, GA; 2 St. Erik’s Eye Hospital, Karolinska Institutet, Stockholm, Sweden; 3 National Eye Institute, National Institutes of Health, Bethesda, MD

## Abstract

**Purpose:**

Our goal was to improve and standardize the procedure for subretinal injection of mouse eyes. Also, we wished to optimize conditions for electroporation of retinal pigment epithelium (RPE) cells in the mouse eye with naked plasmids.

**Methods:**

Mouse eyes were injected subretinally with reporter plasmid DNA, nanobeads, or buffer. A blunt needle was introduced across the cornea, through the pupil, into the vitreous, until it made contact with the neural retina. Gentle pressure was applied to the needle, forcing it to puncture the retina and enter the subretinal space. Fluid was injected subretinally, raising large blebs evident on the mouse fundus. Subretinal injection surgery and outcomes were monitored and evaluated by video recording. Vidisic^R^ aided in fundus examination of the blebs. Pores in RPE cells, across which the plasmid in the fluid could diffuse, were created by several short electrical bursts. Expression of the delivered gene, *tdTomato*, in the plasmid was examined under fluorescence microscopy to identify targeted cells. Electroporation conditions were varied from 0 to 200 V, 5 to 10 pulses, 0.1 ms to 100 ms duration of each pulse, and a space of 1.5 to 2 mm between electrodes on the cornea and sclera.

**Results:**

A critical sign of surgical success was the appearance and persistence of three large blebs in the mouse eye. This was illustrated by video recordings from two different systems. Application of Vidisic^R^ to the cornea made immediate examination of the fundus possible at the end of the surgery. An 80% success rate was readily achieved by following this method. Once a successful subretinal injection technique was established, electroporation conditions were evaluated. Parameters of 50 V, 1 ms pulse duration, 5–10 pulses, 1 s apart and electrodes spaced 1.5–2 mm apart with the anode placed on the sclera in the vicinity of the blebs produced a tight pattern of RPE transfection at approximately 30% efficiency.

**Conclusions:**

A standardized surgical method and a fast distinct indicator of successful surgery were essential to establishing a gene delivery system based on subsequent electroporation. With the surgery better controlled, electroporation was adequate to transfect a substantial number of RPE cells in a defined position in the globe.

## Introduction

The subretinal space is an excellent target for drug delivery [1-5] and gene therapy purposes [6-21]. This is because subretinal delivery places injected material in contact with the plasma membrane of the photoreceptor (PhR) and the retinal pigment epithelium (RPE) cells, and subretinal blebs formed in that process regress quickly. Subretinal injection surgery is commonly used clinically and has been demonstrated in many animal models. The small size of the mouse eye and the relatively large size of the mouse lens make the surgery more difficult in mice.

Timmers and coworkers [22] described a subretinal injection approach into the eyes of rats via a transcorneal route. Other researchers have used a transscleral, route entering at the limbus or pars plana, crossing through the vitreous, penetrating through the diametrically opposite retina into the subretinal space [23]. Some routes include a transscleral-transchoroidal-Bruch's membrane approach without penetrating the retina [24-26]. These routes are effective for injecting virus, viral particles, liposomes, plasmids, drugs, and formulations, or in collecting the contents of the interphotoreceptor (subretinal) space. However, the small size of the mouse eye and the comparative toughness of the sclera increase the risk of accidentally induced hemorrhages at the ciliary body or during penetration of the choroid. These hemorrhages cause autofluorescence and retinal damage, rendering further treatment or experimentation futile.

External to the RPE cell are four barriers to the delivery of DNA as a therapeutic agent [27,28]: matrices, dilution, degradation, and impermeable membranes. The extracellular matrix of the subretinal space [29] limits convection and diffusion. Dilution reduces the yield of systemically delivered drugs, but is ameliorated by placement of the drug immediately adjacent to the target cell. Drugs may be degraded or destroyed by enzymes [30]. The hydrophobic character of the lipid bilayer prevents hydrophilic DNA from crossing the membranes. If subretinal injections could be made reliable in mice, they would obviate many of these problems by placing DNA immediately adjacent to the RPE cell.

There are several ways to transfect DNA into target cells, among them viral or viral-like vectors, spheroplasts or protoplasts [31,32], liposomes [33,34], physical (electroporation and sonication) [35-38], chemical (DNA compaction [39], dendrimers [40], and precipitates—e.g., calcium phosphate) [41]. Of these, viral vectors approach 100% transduction efficiency in cultured photoreceptor cells [17], and electroporation in the range of 80%–90% transfection efficiency [42]. Other chemical-based agents are highly successful but may require serum-free conditions [43], a state that is impossible in vivo. In this article, we focused exclusively on electroporation as a simple mechanism to deliver DNA to the RPE cell.

Internal to the cell, various barriers prevent the transport of DNA into the nucleus. Entry to the nucleus can only be gained by traversing several subcellular compartments, each with specific barriers to diffusion, movement, and permeability. The first major barrier is the sorting mechanism by which cells separate internalized materials into degradative pathways in lysosomes or the proteosome. Defenses against viral or bacterial attack [44] act as barriers to therapeutic vectors. The nuclear membrane and nuclear pore complex block entry of large molecules that lack nuclear localization signals. Once in the nucleus, episomal DNA can be inactivated [45]. Finally, the cell can undergo apoptosis as a last resort against delivery of drugs or DNA [46]. All these barriers must be overcome for efficient gene therapy. Electroporation circumvents several barriers by delivering DNA directly across the plasma membrane into the cytosol. Delivery from the cytosol into the nucleus is diffusion limited following naked DNA delivery by electroporation.

Chalberg and coworkers [47,48] recently demonstrated improved transfection efficiency in chick chorioallantoic membranes and living rabbit eyes by using shorter pulses of higher voltages compared to previously reported electroporation conditions. We took a similar approach. As a second-generation gene therapy strategy, electroporation is inexpensive, easy to replicate, harmless under proper conditions, robust across widely different eye diseases and eye tissues, successful [36,37,42], already in use clinically for drug delivery [49], and rapid [50,51]. We found electroporation to work erratically in mice, because of the delicacy of subretinal injection. In standardizing the method of Timmers and coworkers [22], we found it to consistently work well, once we added an evaluation of the fundus to the procedure.

In this article, we present videos of the surgical technique so that others can more readily learn and repeat our approach and that of Timmers et al. [22]. Given this more reliable surgery, it was possible to test the efficacy of subsequent techniques and treatments performed after the surgery. We were able to optimize electroporation to deliver plasmids to RPE cells. We present these conditions and show high-level reporter gene expression from plasmids in the RPE of the living adult mouse. The expression levels might be sufficient for gene therapy purposes.

## Methods

### Mouse husbandry

Mice were used according to ARVO guidelines and as approved by the Emory Institutional Animal Care and Use Committee. C57BL/6, 129/Sv, and Balb/C, between 1 and 4 months old at time of surgery, were used. Rpe65 knockout mice on a C57BL/6 background were used in several control experiments. Mice were housed at 23 °C in facilities managed by the Emory University Division of Animal Resources and given standard mouse chow (Lab Diet 5001; PMI Nutrition Inc., LLC, Brentwood, MO) and water ad libitum. They were maintained on a 12 h:12 h light-dark cycle, with daytime lighting ranging 200–750 lm outside the cage depending on lower, middle, or top shelf position of the cage rack. After experimentation, mice were euthanized by CO_2_ asphyxiation.

### Dissecting microscope systems

Subretinal injections were performed using one of three different dissecting microscope systems, the first being a Seiler Askania SMC4 stereo binocular microscope (Georgia Instruments, Inc., Roswell, GA) with a halogen fiberoptic ring light (Fiberlite Model 180; Dolan-Jenner, Inc., Boxborough, MA). This system did not include a video camera. The second system was a Wild-Heerbrugg M691 stereo binocular microscope (Leica Microsystems, Bannockburn, IL) fitted with a Hitachi KP-D20BU color CCD video camera (Hitachi, Tokyo, Japan) and a Panasonic DMR-E95H DVD recorder (Panasonic Electronic Devices, Knoxville, TN). The third system was an Olympus SZX2-ZB16 stereo microscope (Hunt Optics; Pittsburgh, PA), which was equipped with a ring light source (Schott; Auburn, NY), and a Panasonic GPUS932HT HD Video camera (Hunt Optics and Imaging) to record subretinal injections. The camera was interfaced to an KONA LHe HD-video capture card (AJA Video Systems, Grass Valley, CA) installed in a Mac Pro (Apple Computer, Cupertino, CA) running OSX Leopard 10.5.3, and the videos were edited with Final Cut Pro (version 6; Apple Computer).

### Surgical equipment and instruments

A NanoFil™ Sub-microliter injection system with a UMP-II microsyringe pump and Micro4 controller with a footswitch was procured from World Precision Instruments (WPI, Sarasota, FL). A heating pad and pump were used to maintain the mice at 37 °C during anesthesia (T/Pump TP500; Gaymar, Orchard Park, NY). Also obtained from WPI were beveled 34 gauge needles (catalog number NF34BV-2) and curved forceps (catalog number 15915). Most other incidental equipment and tools were from Fisher Scientific (Pittsburgh, PA) or VWR (West Chester, PA). Dr. Mann Pharma, Berlin, Germany, distributes Vidisic^R^ Augengel (catalog number 1–19006), a transparent clear ocular hydro-gel, which contains high molecular weight polyacrylic acid. It was the kind gift of Dr. Philipp Lirk, Department of Anesthesiology and Critical Care Medicine, Medical University Innsbruck, Austria.

### Surgical technique

Sterile surgical technique was employed for all surgeries, which were performed between 8:00 AM and 6:00 PM (one hour after lights on to one hour before lights off). Fluid lines, surgical instruments, and needles were sterilized by repeated rinsing with 70% ethanol and sterile water. The 34-gauge beveled needle could be used 15 to 20 times before replacement.

Mice were anesthetized with 80 mg ketamine and 12 mg xylazine per kilogram bodyweight (K-113; Sigma-Aldrich, St. Louis, MO) and achieved adequate anesthesia in approximately 5 min. Each mouse was kept on a 37 °C pad after it was anesthetized, during and after surgery. The cornea was topically anesthetized with one drop of proparicaine (proparacaine hydrochloride ophthalmic solution USP, 0.5%; National Drug Code, [NDC] 17478–263–12; Akorn Inc., Buffalo Grove, IL). The pupil was dilated with one drop of tropicamide (tropicamide ophthalmic solution 1%; NDC 61314–355–01; Falcon Pharmaceuticals, Ft. Worth, TX) and was usually fully dilated within 90 s. If necessary, another drop of tropicamide was placed on the cornea should the pupil not adequately dilate in that time. After dilation, it usually took about 90 s to complete the surgery.

The mouse was positioned with its nose pointing away from the surgeon and its left eye facing up toward the ring light and the microscope. A drop of the carbomer eye gel Vidisic^R^ was placed on the mouse cornea. A 22×40 microscope coverslip was grasped by hand and adjusted on the Vidisic^R^ eye gel in such a way that the fundus, its blood vessels, and the optic nerve head could be easily seen. This served as a means to assess the condition of the eye before injection and as a comparison for the postoperative condition of the retina. The coverslip was removed before the surgery was started.

Mice were not immobilized during the surgical procedure. Instead, after each mouse was placed on a heating pad, its left eye was grasped with curved forceps held in the surgeon's left hand so that the eye was slightly proptosed from pinching of the forceps. A 34 gauge beveled needle, held in the surgeon’s right hand, was used as a lance to make a full-thickness cut through the cornea near the limbus, penetrating upward into the anterior chamber at an oblique (nearly tangential) angle. It was found that some tissue stretching allowed the wound to effectively reseal itself following removal of the needles. The beveled needle was removed and replaced with a blunt 35 gauge needle that was attached to an injection system (WPI). The tip of the blunt needle was advanced into the anterior chamber until it was centered on the optical axis. With a sweeping motion, the needle tip of the needle was moved through the pupil, around the lens, and into the vitreous. While a rare occurrence, if the lens were nicked, the surgery was abandoned. There was no evidence of displacement of the lens following surgery, even though it would seem that some of the zonules of Zinn ought to be damaged.

The needle tip was advanced to the retina opposite to the puncture site until it was but a few tip diameters away from the optic nerve head. The lens magnified the view of the needle. A gentle amount of pressure (the touch of which must be learned by experience) was applied to penetrate the neural retina into the subretinal space, but not so much that the tip penetrated or damaged the RPE sheet. Blood flow into the vitreous meant the tip pressure was too great. A bolus in the vitreous indicated the pressure was not enough and the injection had not filled the subretinal space. The nanojector system was set to deliver 1000 nl at a rate of 170 nl/sec, which allowed for acceptable filling of the subretinal space. It can be useful for an assistant to press the injection button on the face of the nanojector, or alternatively a foot-pedal can be used to activate the microinjector pump. Faster or slower injection rates have not been systematically investigated, but this rate provided acceptable filling of the subretinal space in our hands. The needle was left in position for another 5–10 s to allow pressure in the injection system to equilibrate with the eye pressure and then removed by pulling backwards at a slow pace to allow the hole in the retina to reseal and to avoid damaging the lens, iris, and corneal endothelium during removal. After surgery more of the Vidisic^R^ gel was placed on the eye, and fundus was examined for the presence of blebs. Triple antibiotic ointment (Taro Pharmaceuticals, Inc., Hawthorne, NY), which contains bacitracin, neomycin sulfate, and polymyxin B, was placed on the eye as per ARVO/IACUC guidelines. The mouse was kept on a 37 °C heating pad until it was awake, then it was transferred to recover in a cage by itself for 1 to 2 h. Afterwards, it was moved to its home cage and cage mates until analysis, usually 3 to 4 days after treatment.

### Worksheet

A record of the procedure was kept. Documented were ear number (ear tags from WPI) of the mouse, date of birth, sex, amount of anesthesia, bleb size (small, medium, or large), and number of blebs. Also recorded were any complications including hemorrhage, lens damage, corneal clouding, or the presence of air bubbles.

### Reporter gene plasmid

**Figure 1:**
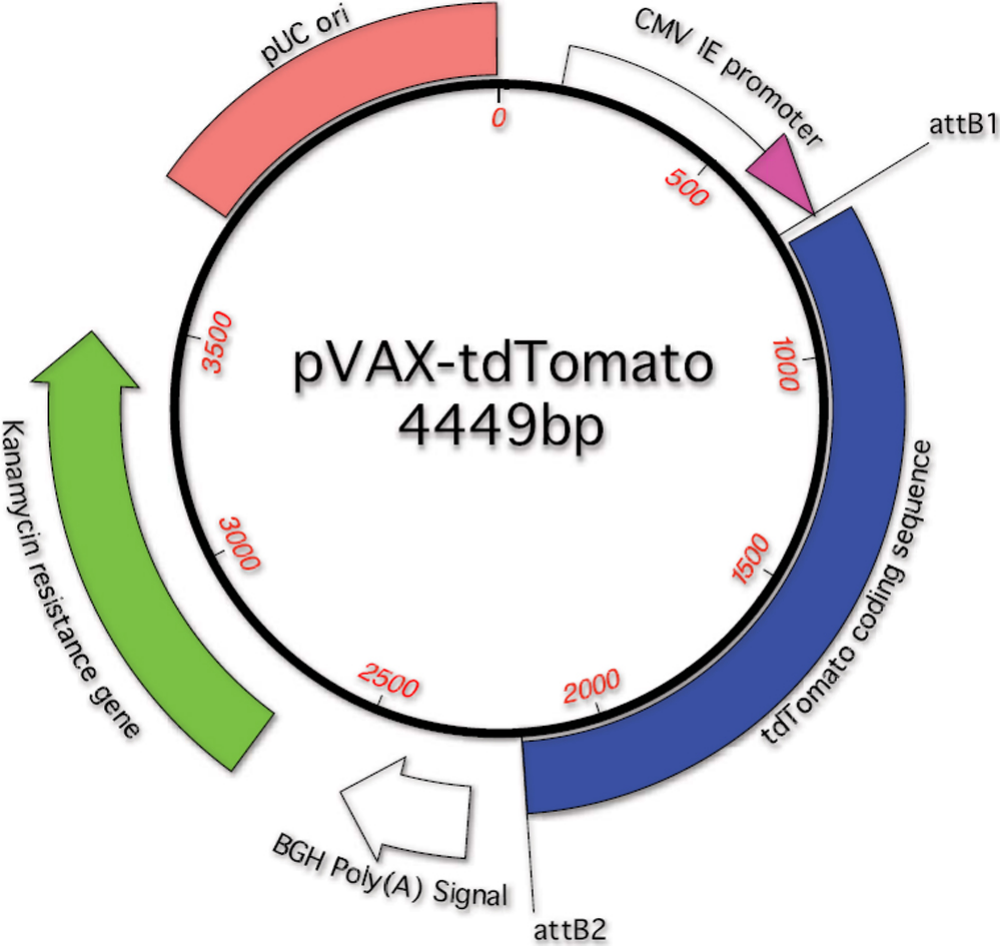
A map of the expression plasmid, pVAX-tdTomato. This expression plasmid was created [52] by inserting tdTomato cDNA into the pVAX™200-DEST plasmid (Invitrogen). The CMV Immediate Early promoter drives expression of tdTomato. Located on the 3′ flanking side of the tdTomato cDNA is the bovine growth hormone poly(A) signal. The plasmid bears the Kanamycin resistance gene, the pUC origin of replication, and attB sites.

The base vector was the mammalian expression vector pVAX™200-DEST (Invitrogen, Carlsbad, CA). *tdTomato* cDNA was PCR amplified from pRSETB-tdTomato with primers bearing AttB sites and incubated with BP ClonaseII and the pVAX™200-DEST plasmid. The resulting reporter expression plasmid, called pVAX-tdTomato [52], contained the CMV Immediate Early promoter driving expression of tdTomato. Located on the 3′ flanking side of the tdTomato cDNA was a bovine growth hormone poly(A) signal. The plasmid contained the Kanamycin resistance gene for selection and growth. A map of the plasmid is shown in Figure 1. This plasmid was a kind gift from Dr. Ton N.M. Schumacher of the Department of Immunology, The Netherlands Cancer Institute, Amsterdam, The Netherlands. Plasmid was isolated from transformed DH10B *Escherichia coli* grown overnight in Luria broth using a Qiagen maxi-prep kit following the manufacturer's protocol. An endotoxin-free kit was not needed for these experiments.

### Sample preparation

Plasmid DNA was resuspended in nuclease-free sterile water at 2 mg/ml. The plasmid solution was centrifuged at 10,000x g for 5 min to sediment any particulates from the solution that might clog the 35 gauge needle. This was done immediately before loading the needle and injection syringe.

Quantum dots with a 600 nm fluorescence emission maximum (EviTags, E2-C11-NF2–0600; Evident Technologies, Troy, NY) were injected as the stock preparation. These dots have a tendency to aggregate, and the aggregates can clog a 35 gauge needle and tubing in the injector system.

### Electroporation

Immediately following subretinal injection, any plasmid-treated mouse eyes or control (vehicle only) eyes were subjected to electroporation. The electroporation source was a commercial square wave generator (BTX model ECM830; Harvard Apparatus, Holliston, MA). Electrodes were made by wrapping platinum-iridium 20 gauge wire (catalog number 50822164; Fisher) around a sharpened pencil tip, creating a 1.5 to 2 mm loop. The loops were clipped to small test-jumper leads (catalog number 278–001; Radio Shack Corporation, Fort Worth, TX) and thence to the BTX electroporator. One platinum loop was positioned directly underneath the retina injection site on the scleral surface of the mouse globe, and the other loop was positioned diametrically opposite from the injection site. The electrodes were spaced roughly 1.5 to 2 mm apart. The loop underneath the injection site served as the positive (anodal) electrode to drive the negatively charged plasmid DNA toward RPE cells.

Optimal conditions and minimum requirements were investigated by varying the voltage, pulse length, number of pulses, and number of pulse trains. An optimum was found with 50 V, 10 pulses, 1 ms pulse duration, 1 s interval between pulses, and one pulse train. The range of conditions tested were: 0.1 ms to 100 ms (0.1, 0.25, 0.5, 1, 10, 25, 50, and 100 ms) for pulse length, 0 to 200 V for potential difference (0, 5, 8, 10, 20, 25, 30, 40, 50, 70, 80, 100, 150, 200 V), 5, 10, and 20 pulses, 0.125 and 1 s interval between pulses, and one or two pulse trains. Typical controls included either omitting plasmid (vehicle-only subretinal injection) or omitting electroporation in different mice. The contralateral eye served as the uninjected control in most mice.

### Analysis of in vivo RPE transfection

A tight pattern of heavy reporter gene expression (as evidenced by fluorescence focused in RPE cells directly over the anode in the RPE sheet) was considered to be an optimal result. Treated and control eyes were harvested from 1 to 14 days after injection. Reporter gene expression was examined by cutting frozen sections of the globes through the center of the eye, the optic nerve head, and the center of the cornea in a superior-inferior plane [53,54] or by creating flatmounts of the entire eyecup [55]. The eyes were fixed in 10% buffered formalin (10% buffered formalin phosphate; catalog number SF100–4; Fisher Scientific) for 30 min on ice, and then rinsed three times in cold PBS for 5 min each.

### Frozen sections

Eyes were harvested following CO_2_ asphyxiation of the mouse. The intact eye was fixed for 30 min in ice-cold 10% buffered formalin (Fisher Scientific), rinsed for 5 min three times in ice-cold Dulbecco’s PBS (dPBS; product number 14200; Invitrogen), and transferred to 20% sucrose in dPBS. After about 15 min, the eye began to sink, and it was dipped three times in a 1:1 mixture of 20% sucrose:tissue freezing medium (Jung; Leica Microsystems, order number 0201 08926, distributed by Vashaw Scientific, Inc., Norcross, GA). The eye was oriented in a cryomold containing 0.5 ml tissue freezing medium and frozen slowly on a plastic canoe floating on liquid nitrogen, and stored at −80 °C until sectioning. Samples were cut at 8 μm; every fifth section was taken on a Leica CM1850 cryostat set to −22 °C (Leica Microsystems). Sections of the globes were cut through the center of the eye, the optic nerve head, and the center of the cornea in a superior-inferior plane [53,54,56] and collected onto Fisher superfrost microscope slides. Slides were stored at −80 °C until staining.

### Nuclear staining

For DAPI staining of nuclei, slides were removed from the −80 °C freezer and dried under a fan for 30 min. Slides were dipped in dPBS, and about 25 μl Vectashield mounting medium with DAPI (catalog number H-1200; Vector Laboratories, Inc., Burlingame, CA) were added, and a coverslip placed on top. For Yo-Pro-1 (Invitrogen) staining of nuclei, sections were air-dried and incubated with 4 μM Yo-Pro-1 stain (diluted with dPBS from a stock solution of 1 mM in DMSO) for 1 h, then mounted in Vectashield Hardset (H-1400; Vector Laboratories). Slides were then examined under confocal microscopy.

### Antibody staining of RPE65

The mouse antibody PETLET [57] was used at a 1:500 dilution in 1% BSA and dPBS. Sections were treated at 4 °C overnight. The primary antibody was washed off three times in dPBS, and a rabbit anti-mouse secondary antibody labeled with AlexaFluor-488 was applied at a dilution of 1:2000 and incubated at room temperature for 2 h. The excess secondary antibody was rinsed off with three changes of dPBS, and the section was mounted in Vectashield Hardset (Vector Laboratories). Rpe65 knockout mouse eye sections were used as negative controls with this antiserum.

### Confocal microscopy

Confocal microscopy was performed using a Nikon Ti inverted microscope equipped with a C1-plus confocal scan head and filter-based detector and 402 nm diode, argon gas, 561 nm diode and 638 DPSS lasers (Nikon Instruments, Melville, NY). The software used to capture the images was Nikon EZ-C1 Gold version 3.60. DAPI was excited using a 402 nm laser line, and emissions were filtered using a 450/35 and 675/50 dual bandpass filter. TdTomato was excited using 561 nm laser line and emissions were filtered using a 605/75 bandpass filter. AlexaFluor-488 (Invitrogen) and Yo-Pro-1 (Invitrogen) fluors were excited using the argon gas 488 nm laser line and emissions were filtered through a 515/30 bandpass filter.

### Apoptosis detection

Cells were stained for detection and quantification of apoptotic cells. A DeadEnd TUNEL kit (product number G3250; Promega, Madison, WI) was used according to the manufacturer’s instructions.

### Flatmounting

The eyecup flatmount included all the cornea and sclera, the neural retina and uveal tract, but the lens was removed [10,58]. A puncture was made in the cornea with a 27 gauge needle, and iridectomy scissors were used to make four to six radial cuts, starting at the center of the cornea, and extending toward the optic nerve. If necessary, a fresh razor blade or disposable cryotome blade was used to extend the initial cuts started with the iridectomy scissors. The flattened eyecups were placed on microscope slides in 100 μl of dPBS. This solution kept the tissue moist before and in between staining steps. These staining solutions were pipetted on and off with handheld pipettors, but otherwise did not differ from staining procedures for frozen sections.

### Fluorescence detection of reporter gene expression

Detection of reporter gene expression was conducted by fluorescence microscopy. The *tdTomato* reporter gene has an excitation optimum at 395 nm and a lesser peak at about 460–490, an emission maximum at 581 nm. Compared to other naturally fluorescent proteins, tdTomato has reduced photobleaching and provides excellent fluorescence [59]. Epifluorescence microscopy was used to detect this red fluorescence. For the photomerged flat mounts, we used an inverted microscope (Diaphot 300; Nikon Instruments) with a triple-bandpass filter cube (DA/FI/TX-B; Semrock Inc., Rochester, NY) and a ProgRes CF CCD camera (Jenoptik Laser Optik Systeme GMBH, Jena, Germany). When we found an area of interest, we used the aforedescribed confocal system to obtain a detailed image of the RPE cells expressing the tdTomato.

## Results

### Basic subretinal injection procedure and videos

**Figure 2:**
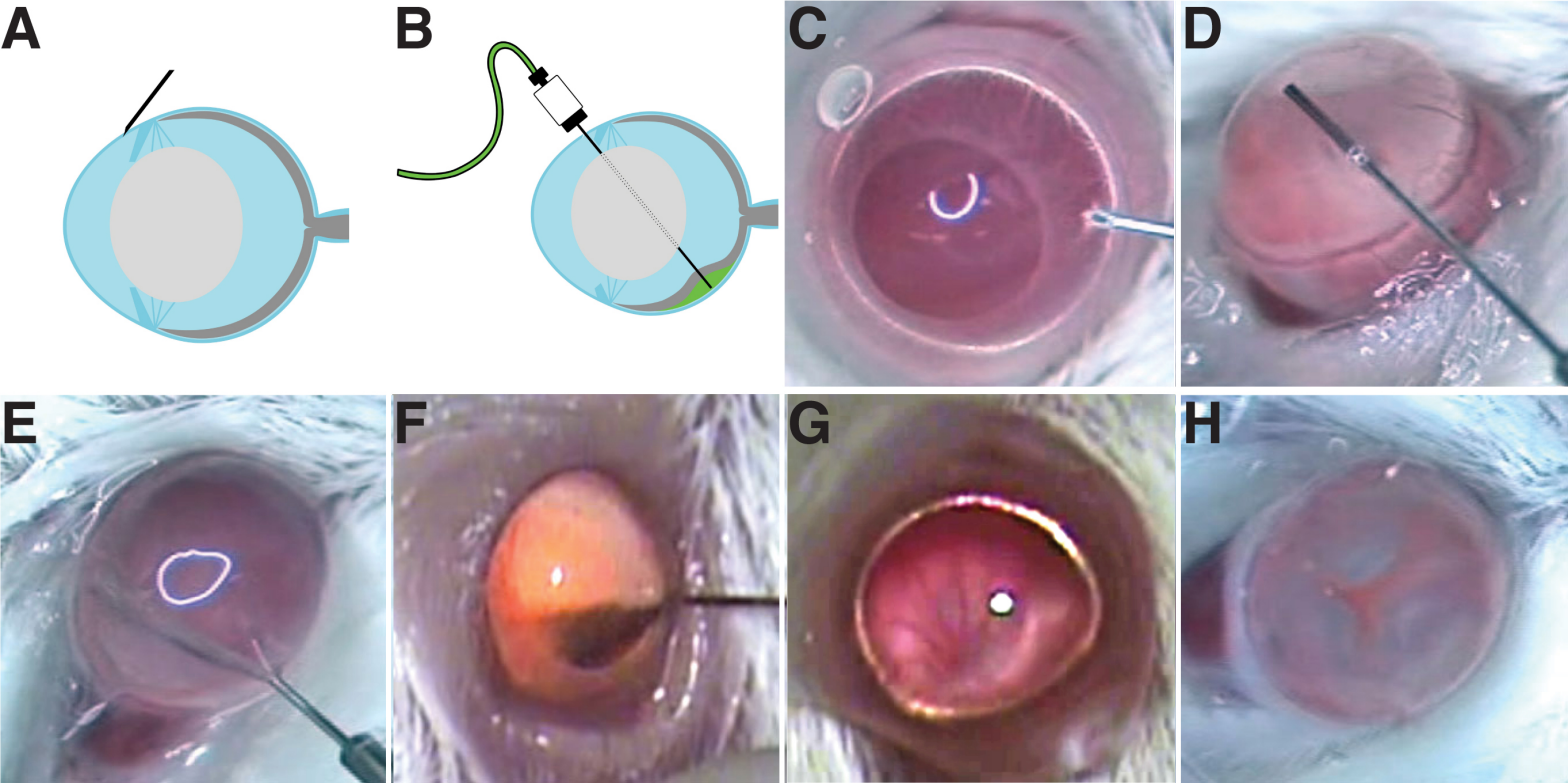
The subretinal injection technique. **A**: Position of the 34 gauge beveled needle is shown nearly tangential just before lancing the cornea. **B**: This schematic illustrates the position of the 35 gauge blunt needle after puncturing the neural retina and partially inflating the interphotoreceptor space (the subretinal space) to produce subretinal blebs. **C:** Presented is a still image from a video illustrating penetration of the cornea. **D**: This panel shows the positioning of the 35 gauge blunt needle in the center of the anterior chamber. **E:** The 35 gauge needle penetrates through the retina into the subretinal space. **F:** The 35 gauge needle is removed from the vitreous after subretinal injection of quantum dots. A small number of quantum dots are evident in the vitreous that generate a reddish-orange color. **G**: Illustrated is a fundus before subretinal injection. The retinal vessels can be readily detected in the fundus image. A ruddy red background color can be observed before injection. **H**: Shown is the fundus immediately after subretinal injection. The positions of three blebs surrounding the optic nerve head are located at clock face positions 4, 8, and 11. Each bleb appears puffy and gray in color with red vessels between the blebs. The optic nerve head is nearly centered in the image of the fundus. The imaged mouse eyes are about 3 mm in diameter.

**Figure 3:**
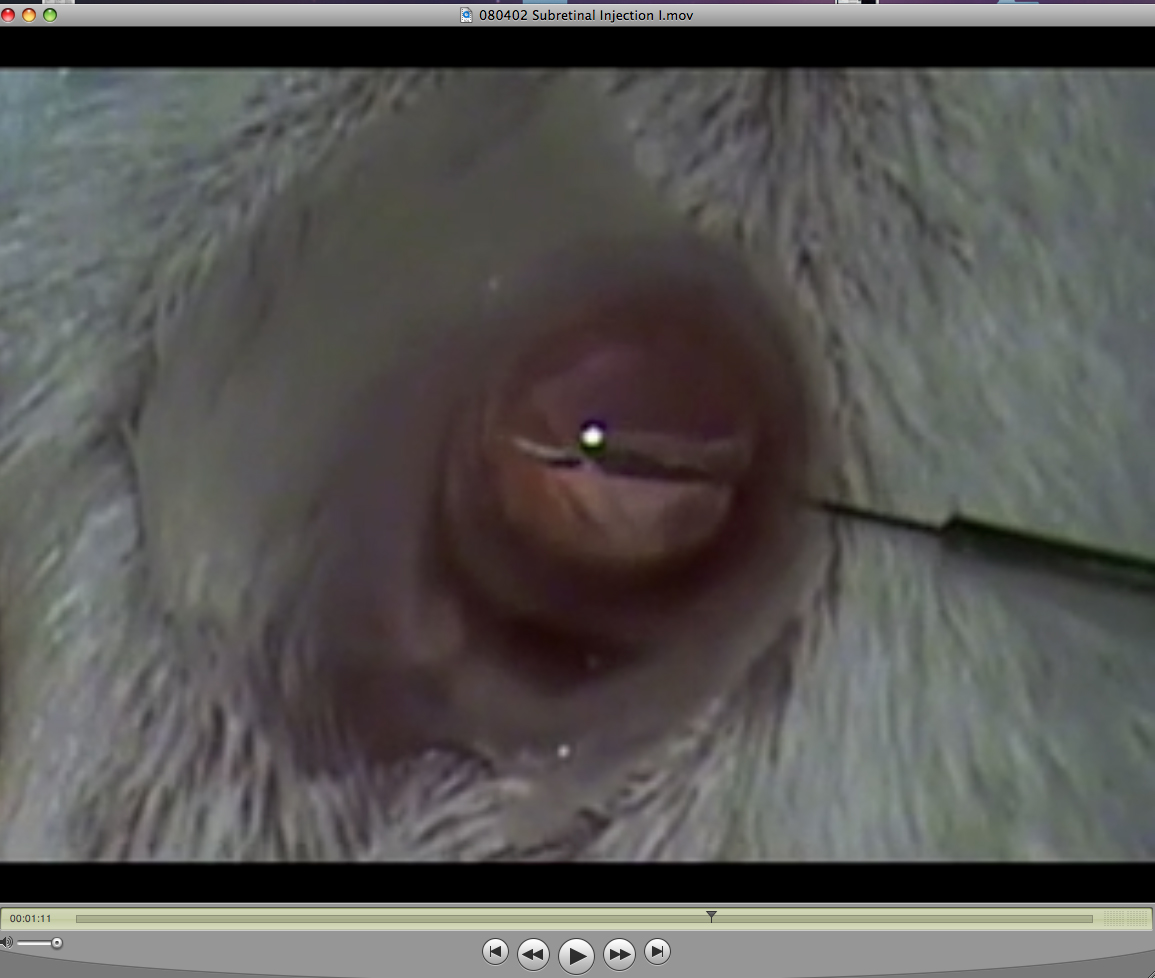
Subretinal injection by transcorneal route. This video was created on a Wild-Heerbrugg dissecting microscope equipped with a ring light and a standard definition CCD video camera. Double-click on the image to play the video. Note that the slide bar at the bottom of the quicktime movie can be used to manually control the flow of the movie. If you are unable to view the movie, a representative frame from the movie is included. This image illustrates the transcorneal puncture.

**Figure 4:**
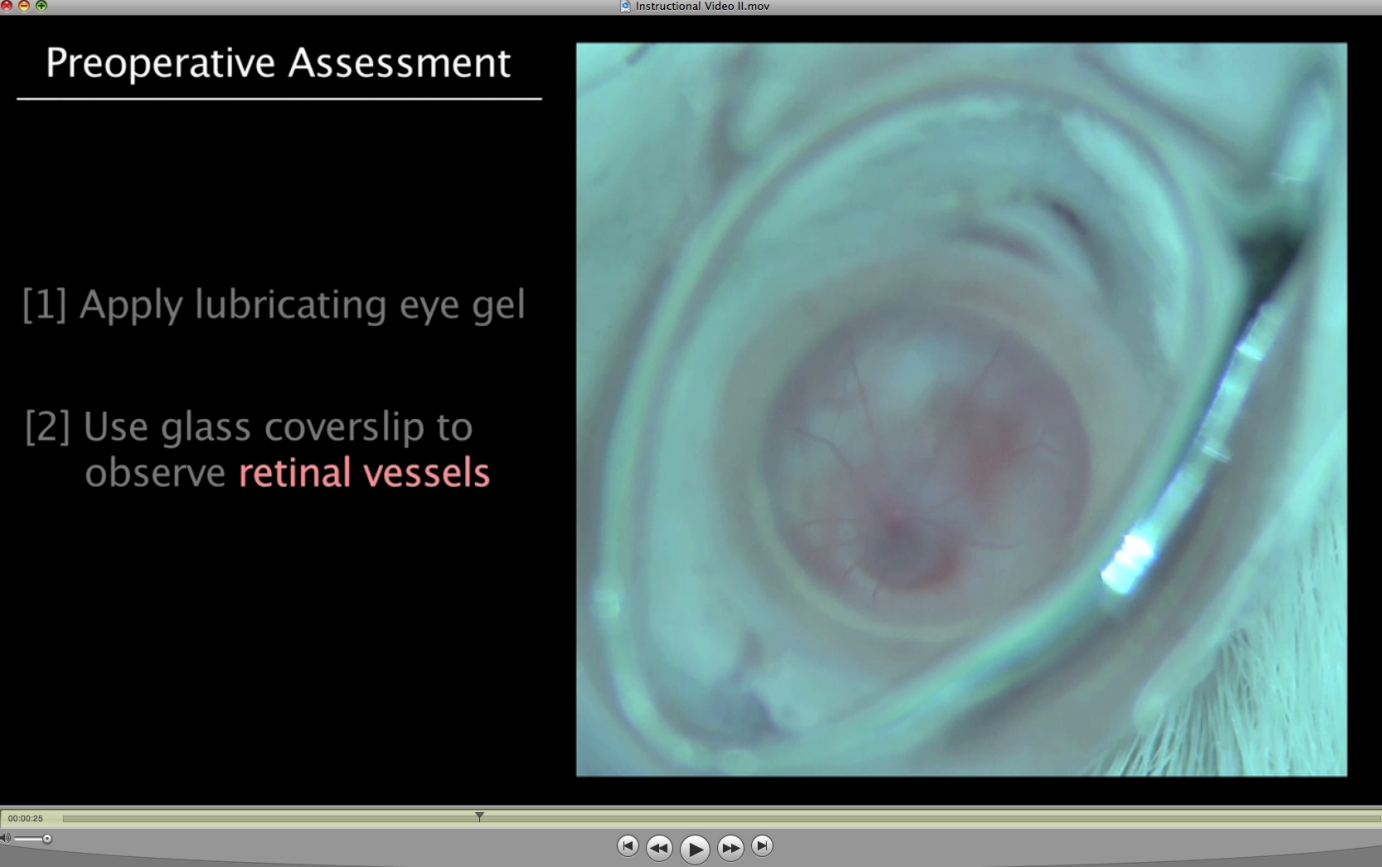
Subretinal injection by transcorneal route. This video was created on an Olympus dissecting microscope equipped with a ring light and an HD video camera. Double-click on the image to play the video. Note that the slide bar at the bottom of the quicktime movie can be used to manually control the flow of the movie. If you are unable to view the movie, a representative frame from the movie is included. This image illustrates the transcorneal lance.

Figure 2 provides static images of the subretinal injection technique. Two videos of the surgical procedure are illustrated in Figure 3 and Figure 4. These videos were made with two separate dissecting microscopes and two different video cameras that allow a comparison of contrast, illumination, and depth of field. These videos and images (Figure 2, Figure 3, and Figure 4) illustrate the successful surgery and injection procedure, highlighting the correct techniques.

### Potential pitfalls

The costs (in time, effort, and mental energy) associated with optimizing this procedure can be significant. Table 1 presents some of the problems we encountered with the injection technique and solutions we found.

**Table 1:** Troubleshooting guide for subretinal injection.

Problem	Probable cause	Solutions
Lens cloudy	Lens capsule nicked during surgery	Avoid the lens.
No blebs	Not penetrating the retina	Press a little harder.
No blebs	Torn retina or retina hole; fluid leaks out quickly into vitreous	Penetrate retina in a single motion.
No blebs or a poorly inflated bleb	Fluid leaks out quickly into vitreous	Pause for 5–10 s before removing the blunt needle from the subretinal bleb. This allows pressure equilibration.
Blood	Penetrating into the choroid	Press gently.
Blood	Nicking the ciliary body	Sweep closer to lens.
Cloudy cornea	Eye was not kept moist before surgery	Apply lubricating eye drops between proparicaine and tropicamide application.
Air bubbles	Air lines or solutions not degassed	Degas solutions. Flush the lines and prime them with water before filling with delivery solution.

This table summarizes a few of the common problems that have been encountered while attempting subretinal injection of the adult mouse eye.

### Signs of a successful procedure

Careful examination of the adult mouse fundus, after surgery, was an easy way to evaluate the success or failure of the injection. In most cases, the fundus could be observed directly through the dissecting microscope after removal of the injection needle, but on occasion a little more Vidisic^R^ needed to be applied to the injected eye, or a coverslip had to be placed in contact with the gel.

Three blebs underlying the neural retina was a sign of proper inflation of the subretinal space in the adult mouse eye. These blebs were clearly visible, and they demonstrated that the injected material was well confined within the subretinal space. We did not observe any cases of more than three blebs being formed. Eyes with only one or two blebs were usually accompanied by evidence of a torn or damaged retina, as seen on funduscopic examination. Mice without three blebs were excluded from experimental test groups, and the injection was considered unsuccessful. There are several causes of incorrect inflation of the subretinal space: 1) material was injected into the vitreous; 2) fluid rapidly leaked out of the subretinal space into the vitreous through a retina hole; 3) retina was hopelessly torn; 4) material was injected elsewhere; 5) pump did not operate correctly; 6) needle was clogged; or 7) material was injected suprachoroidally or subchoroidally. Evidence of blood in the vitreous or aqueous, or nicking of the lens were other indicators of problems, and mice with these were rejected as well. All signs could be readily observed during fundus examination.

### Quantum dots

**Figure 5:**
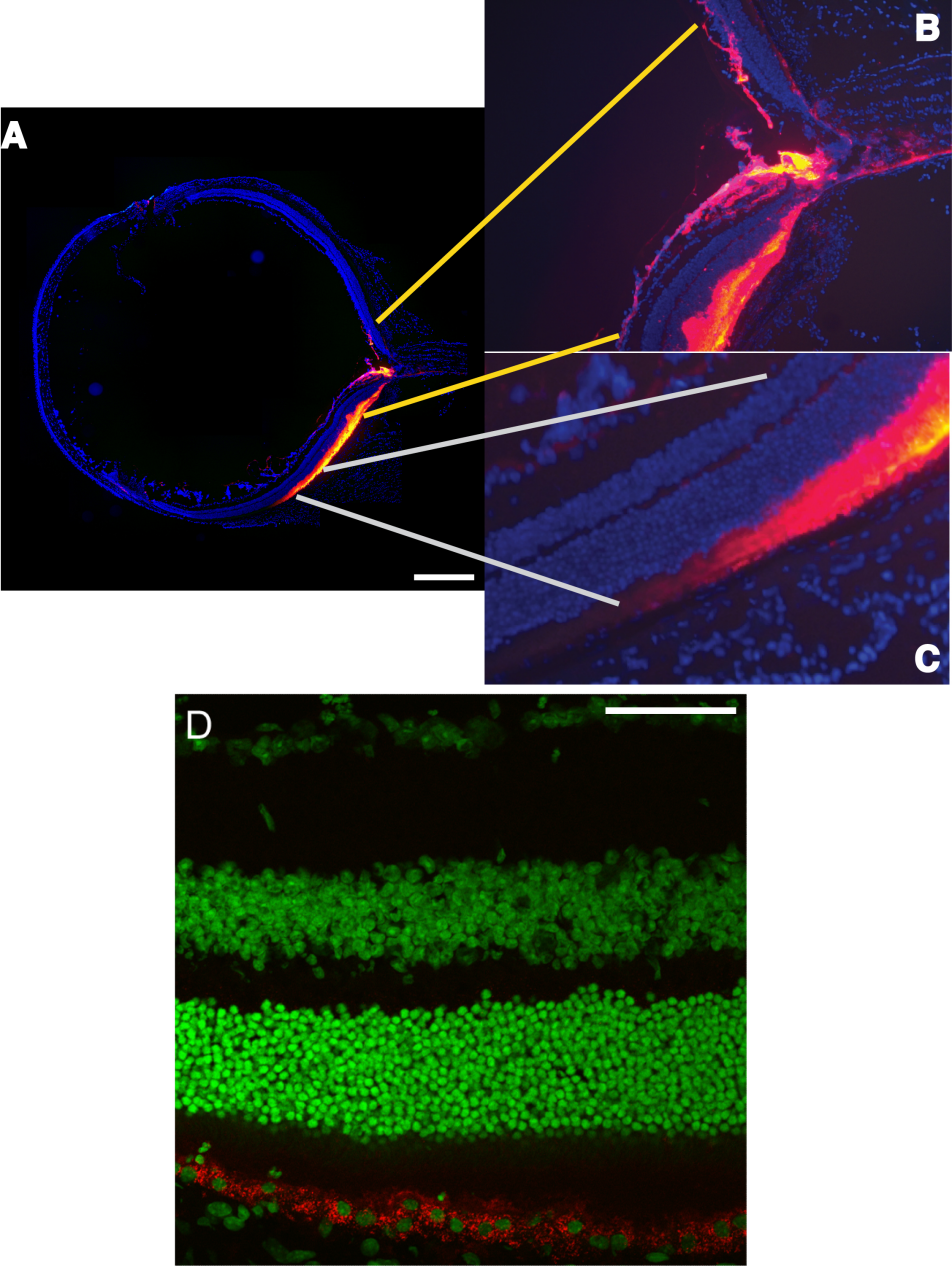
Quantum dots in the whole eye after subretinal injection. The mouse was sacrificed 24 h after injection of the quantum dots. This length of time allowed the three blebs to resorb and the neural retina to return to contact with the RPE sheet. Cryosections were collected and counterstained with DAPI (to stain all nuclei blue). **A**: Shown is a composite of numerous images originally collected with a 20X objective. Images were fused in Photoshop with the Photomerge tool (Adobe Systems Inc., San Jose, CA). The cornea was lanced at clock face position 11, and a small number of quantum dots can be seen in the corneal stroma at the wound site. The retina was punctured between clock face positions 4 and 5. The bright yellow represents intense fluorescence of highly concentrated quantum dots located in the subretinal space. There is a continuous gradient of color from bright yellow to purple, indicating successively less fluorescence of the quantum dots in proportion to their concentration. The quantum dots were confined specifically to the subretinal bleb. The scale bar represents 500 μm. **B**: A few dots were found in the optic nerve head and interstitial spaces in the optic nerve, as indicated by the red and purple colors. **C**: A distinctive gradient of color from bright yellow to purple is illustrated from right to left within the confines of the subretinal space. The comparative absence of any color other than blue-stained nuclei in the section suggests that there was no break in the RPE sheet or tear in the neural retina during subretinal injection. **D**: Close-up reveals quantum dots phagocytosed within the RPE cells. Quantum dots (red) were detected at the level of the RPE cells, and the dots surround the Yo-Pro-1 stained nuclei (green) of the RPE cells. This location indicated that the quantum dots were internalized into the RPE cells. The scale bar represents 50 μm.

The boundaries of the injection site were mapped with nondegradable nanoparticles that were injected into the subretinal space. The blebs raised by injection regressed within 24 h, and the eyes harvested. Frozen sections were taken to examine whether the quantum dots were confined to the subretinal space (Figure 5). A great amount of red fluorescence was present in the subretinal space. Only small numbers of quantum dots were seen in locations other than the subretinal space, suggesting little leakage from the site of injection. These included small numbers in the optic nerve and a few dots near the initial puncture in the cornea. A small number of dots were found in the vitreous. In the location of the resorbed bleb, the outer nuclear layer, inner nuclear layer, and ganglion cell layer all exhibited qualitatively normal thicknesses. Figure 5D shows a close-up of the retina, subretinal space, and the RPE. The quantum dots (red fluorescence) seen in this image were apparently internalized (perhaps by phagocytosis and endocytosis) into the RPE cells. The nuclei had been stained with Yo-Pro-1, which stains nuclei green (Figure 5D). These experiments with quantum dots established the boundaries of the subretinal injection site, a comparative absence of leakage out of the bleb, and provided a long-term marker of the deposition site.

### Optimized expression of tdTomato

**Figure 6:**
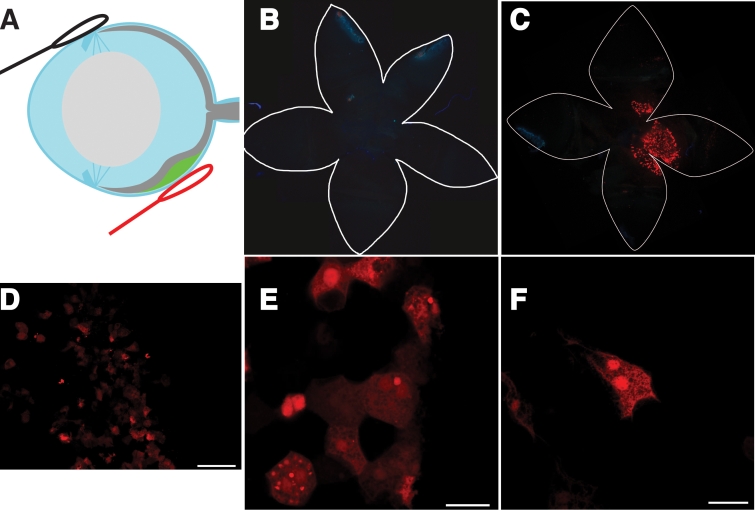
Expression of a reporter gene in RPE cells; subretinal injection and electroporation under optimized conditions. Red fluorescence of tdTomato expression was observed following subretinal injection and electroporation under optimized conditions; individual RPE cells were resolved. The cells include neighbors with and without tdTomato fluorescence (red). About 30% of the cells in electroporated area were positive for tdTomato gene expression. This field represents the RPE cells located directly over the anode. TdTomato-expressing cells exhibited polygonal shapes characteristic of normal RPE cells. The electroporation conditions employed were 50 V, 2 mm gap between electrodes, 1 ms pulse duration, and 10 pulses at 1 s intervals. **A**: In this schematic of the eye, the bleb is in green and the positive electrode in red. The black electrode represents the negative electrode. **B**: Presented is a wholemount of the eye after bleb formation but without voltage applied to the electrodes. The edges of the flatmounts are outlined in white and form a floret shape. The center of the floret corresponds to the retina while the outer half of the “petals” correspond to the cornea. **C**: Shown is a wholemount of the eye following subretinal injection and electroporation under the optimized condition. A focused patch of red fluorescent cells is evident near the center of the floret. Each dot represents a separate RPE cell. This region of the retina corresponds to the bleb and the location of the anode. **D**: High magnification of the fluorescent region shows about 30% of the RPE cells manifesting tdTomato fluorescence. **E**: Close-up of a cluster of tdTomato fluorescence in cells reveals a cobblestone or polygonal shape. **F**: Shown is a close-up of a single binucleate RPE cell. In **B** and **C**, images are about 9 mm across. The scale bar in **D** represents 50 μ. The scale bars in **E** and **F** represent 25 μm.

**Figure 7:**
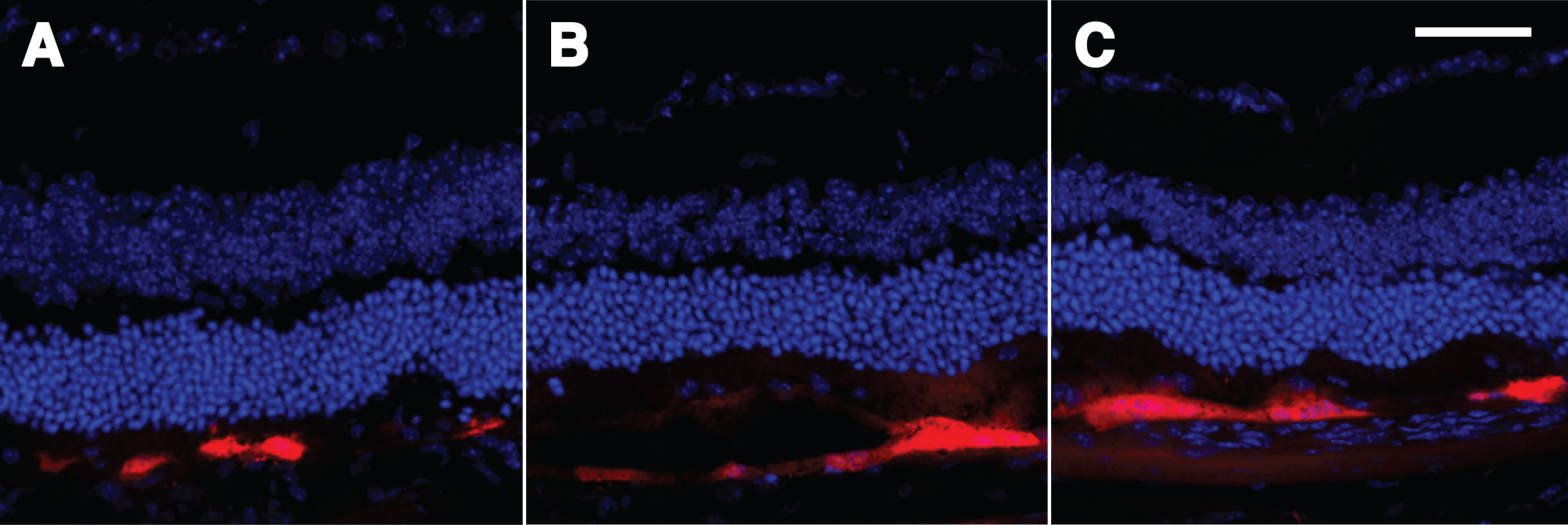
Electroporation of the mouse eye viewed in cross section. **A**, **B**, and **C**: Demonstrated are the results of representative experiments with optimized electroporation conditions after the subretinal injection of pVAX-tdTomato [52]. The cross-sections were cut with a cryostat and nuclei stained with DAPI. **A**, **B**, and **C** all illustrate specific expression of tdTomato in the RPE cell layer. The red RPE cells each appear to subtend a region of about 50 nuclei in the outer nuclear layer, consistent with the known relationship of each RPE cell supporting numerous PhR cells. The scale bar represents 50 μm.

Once standardized conditions for the subretinal injection were established, it was possible to systematically examine the efficacy of gene delivery by electroporation into the RPE cells in the adult mouse eye. In Figure 6, we illustrate the technique and optimal electroporation conditions. Figure 6C shows a tightly focused cluster of RPE cells that express tdTomato. Close-ups (Figure 6D) of this tight patch revealed about 30% of the cells were red. The polygonal pattern of RPE cells was observed in both transfected and nontransfected cells, and there was no characteristic bias in the transfection of cells of lesser or greater polygonality. Figure 6E,F show the red fluorescence illuminating polygonal or cobblestone-like cell borders, characteristic of RPE cells. Cross-sections (Figure 7) revealed the red cells to be in the RPE cell layer and to be the size of RPE cells, subtending about 50 nuclei in the outer nuclear layer.

**Figure 8:**
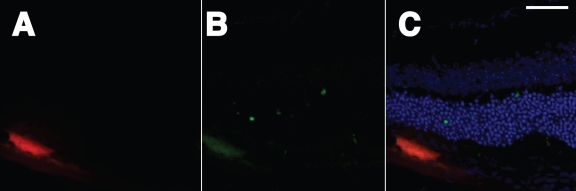
Expression and death assessment in the retina following subretinal injection and electroporation of pVAX-tdTomato. **A**: Cross-sections of the retina were cut with a cryostat. Red fluorescence was detected in a single cell found in the RPE layer. This image is the red channel from a confocal image. **B**: TUNEL staining (green channel) was done in the same section. This panel shows three TUNEL-positive nuclei in a field of roughly 400 nuclei in the PhR cell layer in the image. **C**: **A** and **B** were merged and show the DAPI channel (blue) of the same section. The scale bar represents 50 μm.

Tissue death under these optimized conditions was examined (Figure 8). A limited number of apoptotic cells were detected as reflected by punctate green fluorescence in the outer nuclear layer in cross-sections. No green nuclei were detected in the RPE cell layer.

**Figure 9:**
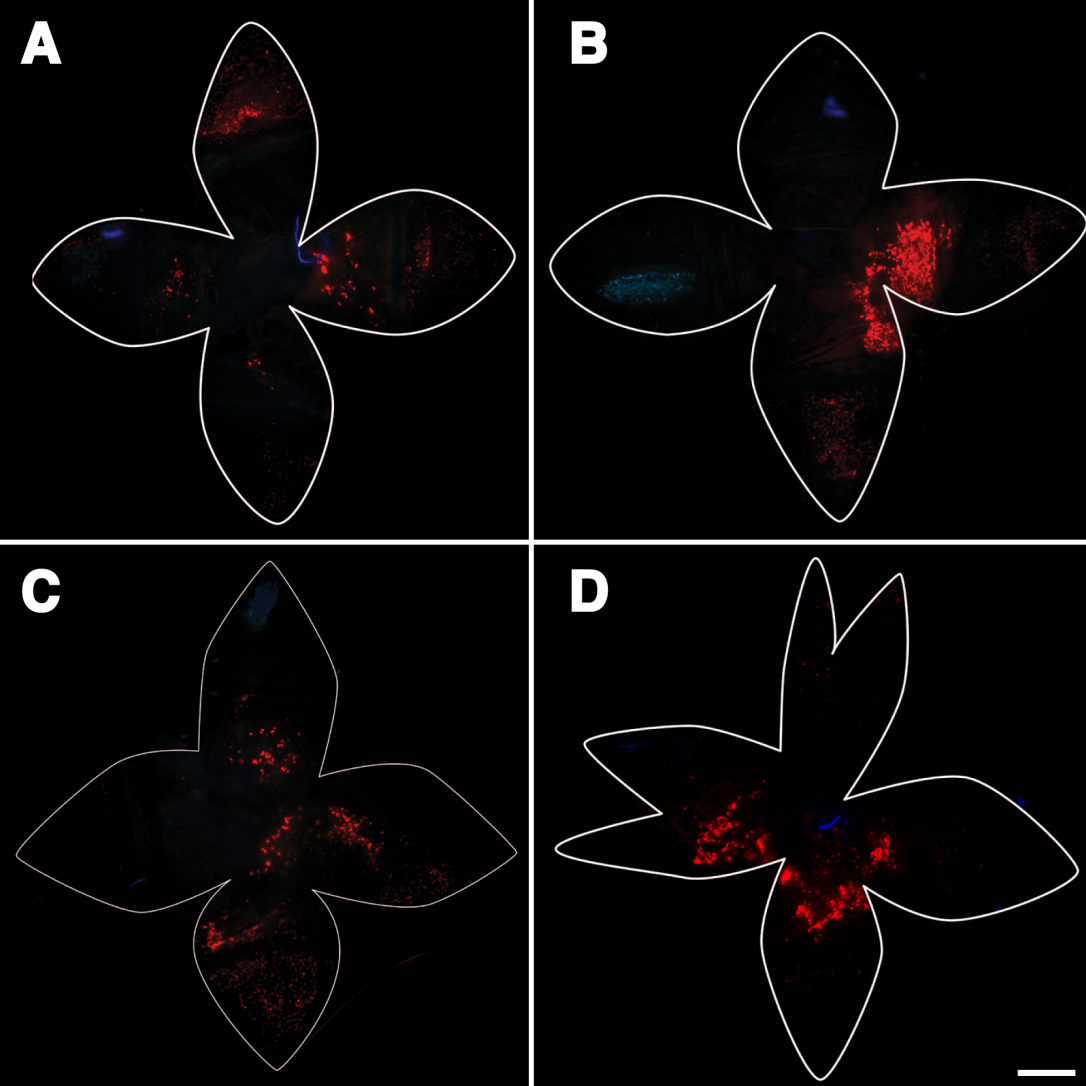
Subretinal injection followed by electroporation, and analysis of flatmounts. Four different electroporation programs were employed to compare qualitatively their efficacy. These conditions resembled the voltage, pulse duration, and train of four different published techniques to electroporate the retina and RPE sheet in other species and at differing stages of development. **A**: The conditions were 30 V, 8 pulses, 50 ms duration per pulse, and an interval of 0.1 s between pulses [61]. **B**: The conditions were 38 V, 5 pulses, 50 ms duration, and 1 s between pulses [62]. **C**: The conditions were 50 V, 5 pulses, 50 ms pulse duration, and 0.95 s between pulses [63]. **D**: The conditions included two voltage steps per cycle. The first voltage was 150 V for 0.25 ms and then 5 V for 5 ms. This combination of voltages was applied 5 times [47]. In all four images, cells were transfected and expressed tdTomato in the flatmount. In most cases, tdTomato accumulated in cells of the cornea, ciliary body, and to a somewhat lesser extent in the RPE. In **A**, **B**, and **C**, there is evidence of red fluorescence in the corneal endothelium. Some of the expression was off center from the position of the anode. In one case there was some evidence of burn damage. The scale bar represents 1 mm.

**Figure 10:**
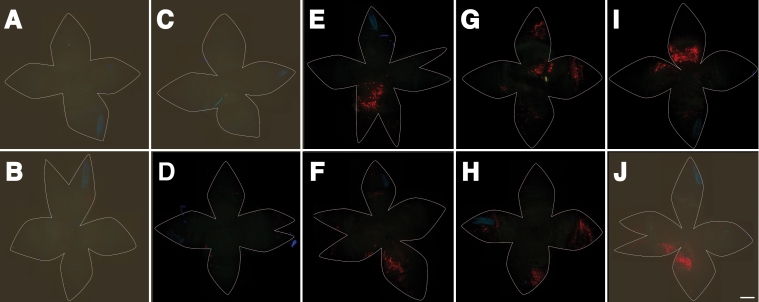
Subretinal injection followed by electroporation at different voltages. In all panels 1 μl 2 mg/ml pVAX-tdTomato in water were injected and three blebs were raised. Five different voltage settings (0, 25, 40, 70, and 100 V) were used. Electroporation followed with a constant 1 ms pulse, 1 s interval, and 5 pulses on panels **A**, **C**, **E**, **G**, and **I**, and 10 pulses with panels **B**, **D**, **F**, **H**, and **J**. In **A** and **B** the samples received 0 V, in **C** and **D**, 25 V, in **E** and **F**, 40 V, in **G** and **H**, 70 V, and in **I** and **J**, 100 V. Each panel represents a flatmount of the eye excluding the lens. The tips of the florets correspond to the center of the cornea, and the central area from the midpoint or greatest bulge of each petal inwards corresponds to the RPE sheet. The red fluorescence is punctate; each dot corresponds to a single RPE cell. When no voltage was applied, there was no evidence of tdTomato expression (red). When 25 V was applied, there was no expression of tdTomato; however, when 40 to 100 V were applied, there was an accumulation of tdTomato fluorescence. The scale bar represents 1 mm.

Several parameters of electroporation were varied to deliver pVAX-tdTomato and to measure its expression level (Figure 9 and Figure 10). Conditions resembling those previously used in other electroporation studies for other species, and for other ages of mice, were employed (Figure 9). All these conditions revealed expression of tdTomato in the RPE with some expression in the cornea, ciliary body, and iris. In some cases, the expression was widely distributed across the flatmount.

Figure 10 shows the optimization and minimum requirements of voltage for fixed numbers of pulses and a uniform pulse duration. Up to 25 V was not sufficient to generate any tdTomato expression. A range of 40 to 70 V was sufficient. At 100 V some tissue damage occurred and expression extended into the cornea, ciliary body, and iris. Pulses of 1 ms produced a tightly confined area immediately overlying the bleb and the anode (Figure 10). There seemed to be a higher density of RPE cells that were transfected within this confined area, suggesting that shorter duration pulses helped to create a high level of transfection and lower cell death close to the anode. A third variable was the number of pulses, either 5 or 10. There was little difference between the two at longer pulse durations. It appeared that 10 pulses marked a trend toward more transfected cells under the 1 ms, 50 V condition (Figure 10). More work is needed to discern whether the number of pulses can be further optimized, and as a note of caution, each brand of electroporator may have its own optimal conditions.

**Figure 11:**
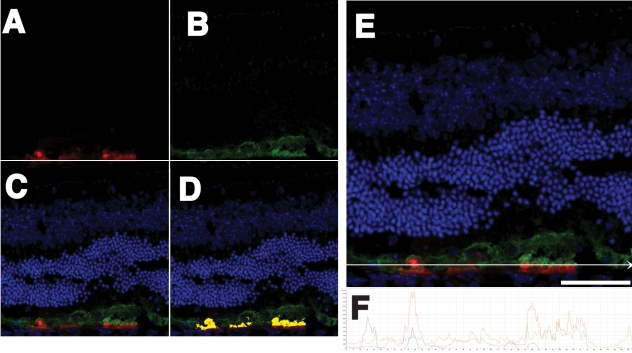
TdTomato expression and RPE65 antibody staining in cryosections. The images show coexpression of endogenous Rpe65 protein (green) and tdTomato (red) in RPE cells following subretinal injection and electroporation of the pVAX-tdTomato plasmid. **A:** Shown is the red channel. **B**: Illustrated is the green channel. **C**: Shown is a composite of the green red and blue (DAPI stained nuclei) channels. **D**: The red and green colocalized in the RPE cell layer (yellow) but not completely. **E**: There were some areas where the green of the Rpe65 protein was observed more apically in the RPE cells and the red of the tdTomato protein was more basolateral and perinuclear. A white arrow drawn across the panel represents an in silico section through which the red and green channels were quantified. **F**: Shown are the relative intensities of red (tdTomato), green (Rpe65), and blue (DAPI) fluorescence in a plot. The intensities follow the white arrow drawn through several RPE cells to scale below the image in **E**. This confocal image was obtained from a mouse eye harvested four days after injection. The RPE cells have survived treatment and coexpress Rpe65 protein and tdTomato in the same cell, though in slightly different locations within the cell. The Rpe65 protein accumulated more on the apical top half of the cell, while the tdTomato accumulated more basally and more in the perinuclear region. The scale bar represents 50 μm.

Figure 11 shows the expression of tdTomato (red) and the localization of Rpe65 protein (green) within RPE cells of wild-type mice. Rpe65 and tdTomato both localized to the RPE cell. Rpe65 protein and tdTomato accumulated in slightly different positions within the RPE cell, with the Rpe65 protein in the more apical half of the cell, and the tdTomato protein found in the more basal and perinuclear regions.

## Discussion

### Improvements to subretinal injection

This technical brief considers some of the difficulties associated with the combination of subretinal injection and electroporation of a naked plasmid. The great variability in outcomes of the subretinal injection in the mouse eye accounted for most of the overall experimental variability in delivery of genes to the PhR and RPE cells when subretinal injection was followed by electroporation. Here we standardized our injection technique and demonstrated our current surgical technique in video form, so that the reader can duplicate and possibly improve upon our present technique. This technique was successful in our hands about 80% of the time, but it still required a significant amount of time to learn. Depending on the amount of practice and prior surgical skills, we found it took 50–100 surgeries to become proficient in the injection of the adult mouse eye. This is in contrast to other researchers’ descriptions of the relative ease [22] and our experience in the subretinal injection of neonatal rat pups, which we found quick and easy to learn. In addition, we found that the microscope itself was not a major variable in the ability to conduct the subretinal injection or observe the fundus. Three microscopes of widely different cost and quality were all used successfully, provided that there was adequate working distance from the mouse to the objective of the microscope.

We found the outcome of the surgery could be rapidly determined postoperatively by examining the adult mouse fundus. The subretinal injection raised blebs, indicating that the retina was elevated from the RPE sheet, and the persistence of these blebs demonstrated the retinal hole had sealed. No bleb meant that the surgery had failed, and the mouse should not be used for further experimentation. We expected only one large bleb, because that is what has traditionally been observed in subretinal injections of neonatal rat pups. However, we found the best outcomes were when there were three blebs, and when the perimeters of these blebs, as viewed by fundoscopy, were demarcated by major blood vessels. Rarely did we observe a single large bleb or two blebs. It is possible that the mouse retina is attached or anchored to the underlying RPE at spots or streaks due to penetrating blood vessels or nerves, and these anchor points may cause multiple blebs instead of one huge one. The blood vessels may be tougher under tension than the more balloon-like, pliant, and weaker neural retina, which can distort its shape more and stretch to inflate the subretinal space between the major arcades of the blood vessels. Several blebs can then be raised, each between the boundaries established by the presence of two stronger blood vessels. It should be noted that bleb formation is also used as a sign for successful subretinal injection in human patients [6,7,9,14].

The use of Vidisic^R^ helped in the rapid evaluation of the fundus. It provided enough optical shape that the fundus was readily apparent. Also, this obviated the need to compress or distort the shape of the cornea with a coverslip, which would put pressure on the delicate subretinal blebs. Eliminating the need for a coverslip increased the percentage of successful surgeries. Methylcellulose was used occasionally, and it is a realistic alternative; however, the Vidisic^R^ seemed to sag less and spread less.

Following subretinal injection quantum dots, there was an absence of fluorescence in the vitreous and intense fluorescence in the subretinal space (Figure 5). After 24 h, the quantum dots were detected at the level of the RPE cells, and the dots surrounded the nuclei of the RPE cells. This location indicated that the quantum dots were internalized into the RPE cells, and likely routes of internalization include phagocytosis and endocytosis. These two routes cannot yet be distinguished; other routes are possible. The route seems unlikely to be pinocytosis as the nanoparticles are likely too large to follow that route. No quantum dots were detected in cells or interstitial spaces in the choroid or sclera, suggesting that there was no interruption to the integrity of the RPE sheet. It is worth noting that to keep the quantum dots dispersed, 1 mg/ml BSA [60] can be added to the suspension, and this prevents clogging of the needle and tubing (a problem that we noted).

After electroporation, tdTomato fluorescence was found in a tight patch of RPE cells nearest to the positive electrode (Figure 6 through Figure 10). No fluorescence was detected at the negative electrode. The positive electrode positioned under the bleb and highly concentrated plasmid are necessary to achieve delivery of the plasmid into RPE cells and expression of tdTomato. Under suboptimal conditions, tdTomato fluorescence was detected in the cornea, ciliary body, and iris. This result occurred with much longer pulse durations (25 to 50 ms).

We found that the tdTomato expression was in polygonal cells characteristic of RPE cells (Figure 6). While there was a variety of patterns of red fluorescence within each cell (with distinctive patterns of speckles, dots, and granularity, some perinuclear and others not) all the cells had a polygonal shape. In addition, we observed that RPE cells coexpress Rpe65 protein and tdTomato in the same cell (Figure 11), though in slightly different locations within the RPE cell. This clearly demonstrated the tdTomato expression to be within RPE cells, which were the cells that we sought to target. The Rpe65 protein accumulated more on the apical top half of the RPE cell, while the tdTomato accumulated more basally and more in the perinuclear region. These patterns may suggest that Rpe65 partitions to the smooth endoplasmic reticulum, which is abundant on the apical side of the RPE cell, while tdTomato may partition to the cytosol. It is not clear why tdTomato expression would be perinuclear. If this protein were to be overexpressed, it might be prone to binding to cytoskeletal elements, or it may become a target of degradative pathways that might compartmentalize it into an aggresome, which has a perinuclear location.

Once most of the experimental variability due to the surgical step was reduced, it was possible to focus more attention on the variability due to changes in electroporation conditions. Our findings, similar to results from Chalberg and coworkers [47,48] in rabbit eyes, suggest that shorter pulses with higher voltages were more efficacious in adult mouse eye than previous findings in vivo and in vitro in different species, tissues, and systems. Our results (Figure 6, Figure 7, and Figure 8) suggest an optimum near 50 V, 5 to 10 1 ms pulses, and about a 1.5 to 2 mm gap between electrodes placed on the surface of the sclera and cornea.

In other preliminary experiments (data not shown), we varied the voltage from 0 to 200 V. A minimum of about 40 V was required before a substantial number of transfected RPE cells were observed. Voltages from 40 up to 80 V demonstrated approximately equal numbers of transfected expressing cells. Voltages beyond about 100 V resulted in immediate evidence of burn damage and were not tested further with pulse lengths at 1 ms or greater. Pulse lengths were varied from 1 ms to 50 ms at different voltages. At 10 ms or longer, the area of the flatmount that showed evidence of transfection extended beyond the immediate location of the electrodes as far as the cornea.

Under less than optimal conditions, in some cases there was less fluorescence in the area over the anode (Figure 9 and Figure 10). This suggested that there may have been tissue damage in the area near the anode, killing transfected cells before they accumulated much tdTomato signal. The tdTomato signal in distant areas such as the cornea might suggest bulk movement of the injected fluid due to rupture of the subretinal blebs, uneven heating, or convection of fluid within the eye that could transport the plasmid to ectopic sites such as the cornea. After such movement, the plasmid could be delivered to these tissues by electroporation. An alternate explanation is that some plasmid is always deposited in these non-target sites and that long (>1 ms) pulses allow electroporation to occur further from the RPE target site.

Further experimentation with even shorter pulses and potentially higher voltages may yield better optimization. However, these standardized experimental conditions are adequate for testing other variables such as the utility of DNA sequences in the plasmid, and other additives that might improve transfection or maintain the stable expression of the transfected gene.
